# Pyoderma gangrenosum induced by transcutaneous electrical nerve stimulation: a case report with literature review

**DOI:** 10.1093/omcr/omac017

**Published:** 2022-03-16

**Authors:** Diana Isabela Costescu Strachinaru, Axel De Greef, Liliane Marot, Valérie Lerate, Marie-Sophie Paridaens

**Affiliations:** Center for Infectious Diseases, Polyclinic Department, Queen Astrid Military Hospital, Brussels, Belgium; Department of Dermatology, Cliniques Universitaires Saint Luc, UCLouvain, Brussels, Belgium; Department of Dermatology, Cliniques Universitaires Saint Luc, UCLouvain, Brussels, Belgium; Department of Histopathology, Cliniques Universitaires Saint Luc, UCLouvain, Brussels, Belgium; Burn Unit, Queen Astrid Military Hospital, Brussels, Belgium; Burn Unit, Queen Astrid Military Hospital, Brussels, Belgium

## Abstract

Pyoderma gangrenosum (PG) is one of the neutrophilic dermatosis, a heterogenous group of rare inflammatory diseases affecting the skin. It is often associated with systemic diseases such as inflammatory bowel disease, rheumatoid arthritis or hematological malignancies. Classical PG is characterized by painful ulcers with violaceous, undermined border, often developing at sites of injury because of the typical pathergy phenomenon. Because of its polymorphic presentation, misdiagnosis and delayed diagnosis are common. We present a case of PG occurring after transcutaneous electrical nerve stimulation (TENS) in a young female patient with ulcerative colitis. Although electric current has previously been incriminated as a trigger for PG, to the best of our knowledge this is the first case precipitated by TENS. We report a typical case of PG occurring after an unusual stimulus and highlight the challenges that the diagnosis of this relatively rare pathology poses to the clinician.

## INTRODUCTION

Pyoderma gangrenosum (PG) is a neutrophilic dermatosis, a rare inflammatory disease affecting the skin [1]. There are several types of PG, including classical (ulcerative), bullous, pustular, granulomatous superficial (vegetative), peristomal and post-surgical [2, 3]. The classical form is the most frequent presentation and occurs mainly on the lower extremities [1–3]. Classical PG is characterized by the development of sterile inflammatory pustules, which expand into painful ulcers with violaceous, undermined borders [1–3]. PG may occur in the absence of an underlying pathology, but in up to two-thirds of cases it is associated with systemic diseases such as inflammatory bowel disease (IBD), rheumatoid arthritis or hematological malignancies [1–3]. IBD is the most common comorbidity in younger PG patients [1, 4]. PG occurs in ~1–2% of IBD patients [4]. Herein, we report a case of PG occurring after transcutaneous electrical nerve stimulation (TENS) in a patient with ulcerative colitis (UC).

## CASE REPORT

**Figure 1:**
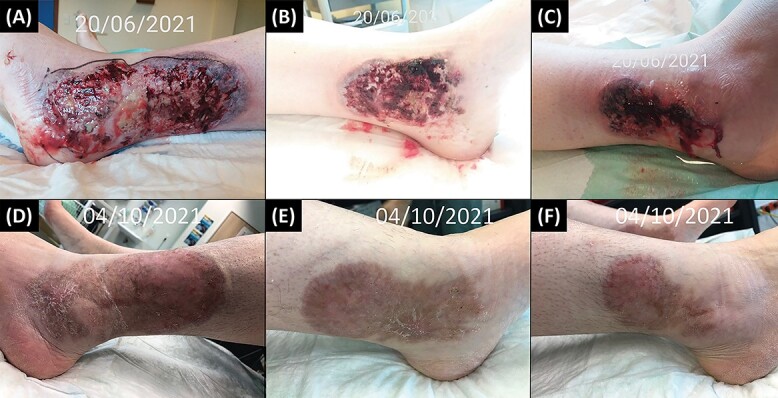
**Panels A–C**: aspect of the wounds at referral to our Burn Unit (**Panel A**: left leg, lateral malleolus, **Panel B**: left leg, medial malleolus, **Panel C**: right leg, lateral malleolus). **Panels D–F**: follow-up at 3.5 months of treatment (**Panel D**: left leg, lateral malleolus, **Panel E**: left leg, medial malleolus **Panel F**: right leg, lateral malleolus).

**Figure 2:**
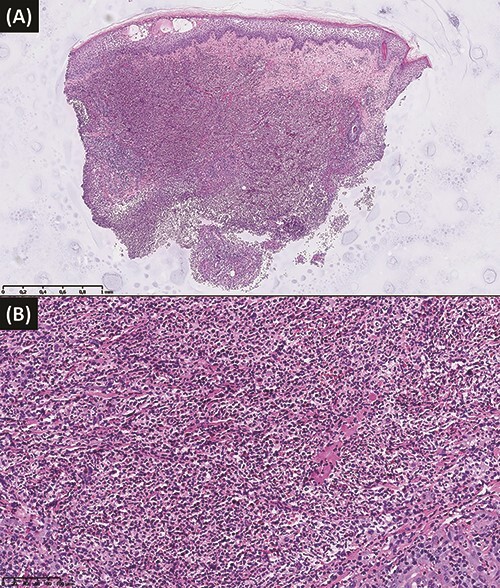
Hematoxylin–eosin stain, showing a dense polymorphic inflammatory dermal infiltrate, very rich in neutrophils (**Panel A**: ×3,46 magnification, **Panel B**: ×20 magnification).

A 27-year-old woman was referred to our Burn Unit for the management of painful, progressive ulcers located on her legs. She had presented bilateral ankle tendinopathy 5 weeks earlier, for which she underwent TENS sessions (mild electrical currents were administered using electrode pads placed on the skin surface of the lateral and medial sides of both ankles). Following the fifth session, bluish-mauve swellings appeared on the left lateral and medial malleoli and on the right lateral malleolus, which ulcerated and extended despite topical cortisone. She reported no fever or systemic symptoms. Daily local bactericidal dressing and successive antibiotic therapies with amoxicillin-clavulanic acid, cefuroxime and piperacillin-tazobactam resulted in the pejoration of the lesions. A computed-tomography scan of the ankles revealed infiltration of the subcutaneous tissue but no bone damage. As there was no improvement, she was referred to our institution with a diagnosis of infected electric burns. Detailed anamnesis revealed a 12-year-old history of UC, stable under mesalazine, not mentioned earlier. Physical examination on admission showed a large purplish ulcer of 16/7 cm, situated on the left lateral malleolus and two smaller ulcers on the left medial and right lateral malleoli (Fig. 1A–C). The rest of the examination was unremarkable. Biology tests were normal except for moderate normocytic hypochromic anemia and a C reactive protein value of 52.8 mg/l (normal range 0–5 mg/l). Immunological (rheumatoid factor, antineutrophil cytoplasmic antibodies, antinuclear antibodies and HLA B27), serological tests (hepatitis B and C, human immunodeficiency virus, syphilis, Rickettsia and Leishmania), bacterial and fungal wound swabs and blood cultures were negative. Skin biopsies revealed a dense polymorphic inflammatory dermal infiltrate, very rich in neutrophils, with a site of deep abscedation, compatible with PG (Fig. 2A and B). Special stains (Periodic Acid Schiff, Wade-Fite, Ziehl-Neelsen and Gram) were negative for bacteria, fungi and mycobacteria. Immunosuppressive treatment with high dose oral methylprednisolone (1 mg/kg/day) followed by the addition of ciclosporin 1 month later, when steroid tapering began, combined with topical tacrolimus and adjunctive hyperbaric oxygen therapy resulted in a favorable evolution, with complete resolution of the lesions and characteristic cribriform scars at 3.5 months (Fig. 1D–F).

## DISCUSSION

PG is the second most frequent dermatologic manifestation of IBD [4, 5]. It occurs mainly when the IBD is active but can also manifest during quiescent periods or precede the IBD diagnosis [4, 5]. Because of its polymorphic presentation, frequent association with various systemic diseases and ability to imitate other conditions, misdiagnosis or delayed diagnosis are common in PG [6, 7]. In our patient, lesions mimicked infected burn wounds, leading to misdiagnosis, inappropriate treatment and clinical deterioration. This case highlights the diagnostic challenges posed by PG and the importance of thorough patient anamnesis, as the patient’s UC history first raised the suspicion of PG.

To this day, PG remains mainly a clinical diagnosis. Skin biopsies are recommended to exclude other causes of cutaneous ulceration. The histopathology of PG typically shows neutrophilic inflammation, but it is nonspecific (infections and other neutrophilic dermatosis may have similar findings) and can vary based on PG subtype, ulcer stage, timing and site of biopsy [7]. To date there is no consensus regarding the diagnosis of PG. Su *et al.* proposed in 2004 a diagnostic tool for classical PG requiring two major and two minor criteria [8], but in this algorithm PG remains an exclusion diagnosis, which can be impractical for clinical decision. In 2018, Maverakis *et al.* proposed new criteria based on the Delphi Consensus of International Experts, requiring one major and four minor criteria, and no longer rendering PG as a diagnosis of exclusion [7]. More recently, Jockenhöfer *et al.* developed another diagnostic tool, the PARACELSUS score [9]. Our patient met diagnostic criteria with each of the three above-mentioned scores.

There is no standardized treatment of PG [2–4]. Topical treatment with steroids or calcineurin inhibitors may be tried in mild forms or those not associated with systemic disease. For systemic treatment, corticosteroids are first-line [2–4]. Corticosteroids may be combined with immunomodulatory agents such as cyclosporine, methotrexate, mycophenolate mofetil or azathioprine. Biologic therapies such as tumor necrosis factor-α inhibitors or interleukin 1 inhibitors have been increasingly proposed in recent years [2–4]. In addition, analgesia and wound care are two cornerstones of PG management. Hyperbaric oxygen therapy can be a helpful adjuvant.

PG lesions may be precipitated by minor traumas, a phenomenon known as ‘pathergy’. Because of this phenomenon, surgery is not recommended as it may worsen the lesions and delay the healing [4]. Although electric current has previously been incriminated as a trigger for PG lesions—Ichikawa *et al.* described PG lesions appearing at the site of the grounding pad of an electric scalpel [10]—this is, to the best of our knowledge, the first reported case of PG precipitated by TENS. We hypothesize that in this case the lesions were triggered by the electrical stimulus in a patient in which the underlying UC put her at a higher risk of developing PG.

In conclusion, we present a typical case of PG occurring after an unusual stimulus, while also highlighting the challenges that the diagnosis and management of this relatively rare pathology pose to the clinician.
